# Population analysis reveals genetic structure of an invasive agricultural thrips pest related to invasion of greenhouses and suitable climatic space

**DOI:** 10.1111/eva.12847

**Published:** 2019-08-08

**Authors:** Li‐Jun Cao, Yong‐Fu Gao, Ya‐Jun Gong, Jin‐Cui Chen, Min Chen, Ary Hoffmann, Shu‐Jun Wei

**Affiliations:** ^1^ Institute of Plant and Environmental Protection Beijing Academy of Agriculture and Forestry Sciences Beijing China; ^2^ Beijing Key Laboratory for Forest Pest Control, College of Forestry Beijing Forestry University Beijing China; ^3^ School of BioSciences, Bio21 Institute The University of Melbourne Parkville VIC Australia

**Keywords:** biological invasion, climatic adaptation, demographic history, population genetic structure, *Thrips palmi*

## Abstract

Biological invasions of pests into climatically unsuitable areas can be facilitated by human‐regulated environments, in which case there may be an impact on genetic structure through population processes and/or adaptation. Here, we investigated the population genetic structure of an invasive agricultural pest, *Thrips palmi*, in China, which has expanded its distribution range through using greenhouses. Early invaded populations showed a relatively higher level of genetic diversity than recently expanded greenhouse populations. Strong population genetic structure corresponded to a pattern of isolation by distance, with no recent gene flow and low historical gene flow among populations, reflecting limited ongoing dispersal. A genetic signature of population expansion was detected in early invaded populations and three northern populations from greenhouses, suggesting that the greenhouse environments facilitated expansion of this species. Redundancy analysis showed that the independent effects of environment and geography could explain 51.68% and 32.06% of the genetic variance, respectively. These findings point to climate‐ and greenhouse‐related spatial expansion, with the potential for adaptation by *T. palmi*. They emphasize the contribution of human‐regulated environments on the successes of this invasive species, a situation likely to apply to other invasive species that use greenhouse environments.

## 1 INTRODUCTION

Spatial spread of many small invertebrate species is often restricted by climatic extremes (Overgaard, Kearney, & Hoffmann, 2014; Wiens & Graham, 2005). However, this restriction can be overcome by species spreading into artificial environments (Bulleri & Airoldi, 2005; González‐Bernal, Greenlees, Brown, & Shine, 2016; Letnic, Webb, Jessop, Florance, & Dempster, 2014) or by undergoing niche shifts due to evolutionary adaptation (Broennimann et al., 2007; Hoffmann, 2017). With the development of greenhouses in agriculture, many pest species raised there have caused heavy damage to plants, such as damage caused by whiteflies, mites, and thrips (Gerson & Weintraub, 2012). Several studies have shown evidence of evolutionary adaptation of species under natural climatic conditions (Csilléry, Rodríguez‐Verdugo, Rellstab, & Guillaume, 2018; Hoffmann, 2017). These factors are not necessarily independent, with many small invertebrates rapidly adapting to controlled environmental conditions when they are reared in the laboratory (Hoffmann & Ross, 2018), suggesting that species might also adapt to controlled greenhouse conditions. The rapid development of insecticide resistance in many pests under greenhouse conditions has previously been documented (Gholam & Sadeghi, 2016), illustrating the potential for evolutionary shifts in such environments.

Genetic variation across populations reflects the effects of population processes like genetic drift under limited gene flow, as well as (at loci under selection or linked to loci under selection) adaptive evolution to local environmental conditions. For invasive species in their introduced areas, population genetic structure can be formed by multiple introductions from genetically differentiated source populations, and demographic events such as bottlenecks and founder effects (Barrett, 2015; Bock et al., 2015; Cao et al., 2017; Konecny et al., 2013; Lee, 2002; Tsuchida, Kudô, & Ishiguro, 2014). Invasive species with a clear population history provide opportunities to examine climatic adaptation through geographic or temporal comparisons (Egizi, Fefferman, & Fonseca, 2015; Hoffmann, 2017; Lee, 2002; Tsuchida et al., 2014; Yamanaka, Tatsuki, & Shimada, 2008), and the effects of human‐regulated environments on the genetic structure of such populations can also be investigated.

Thrips species represent small insects from the order Thysanoptera. Several species of thrips cause serious damage to crops worldwide (Mouden, Sarmiento, Klinkhamer, & Leiss, 2017; Reitz, Gao, & Lei, 2011). The cosmopolitan pest of western flower thrips, *Frankliniella occidentalis* (Thysanoptera: Thripidae), is a temperate species originating from southwestern USA and has invaded other areas around the world since the late 1970s (Kirk & Terry, 2003). It was introduced into China around 2003 and rapidly spread into most areas of China within 10 years. Population genetic analysis suggested multiple independent invasions of *F. occidentalis* into China (Cao et al., 2017). A lack of isolation by distance and the fact that distant populations were genetically similar suggested patterns of movement in this thrips linked to human activities (Cao et al., 2017). Spatial spread of *F. occidentalis* seems not restricted by environmental conditions, which contributes to its cosmopolitan status as a pest and its lack of population genetic structure in introduced areas.

Another important pest thrips, the melon thrips, *Thrips palmi* (Thysanoptera: Thripidae), was first described in tropical regions of Sumatra in Indonesia (Karny, 1925). Early reports of *T. palmi* were exclusively from southeastern and southern Asian countries of Thailand, India, Pakistan, Malaysia, and Philippines (Ananthakrishnan, 1955; Bhatti, 1980). The species was introduced and became established during the second half of the twentieth century across South‐East Asia, South America, the Caribbean, Florida, Australia, and West Africa (Cannon, Matthews, & Collins, 2007; Kawai, 1990, 2001). Climatic conditions are thought to have restricted the spatial expansion of *T. palmi* (McDosald, Bale, & Walters, 1999). In northern Japan, this species could not overwinter outdoors, while in Australia it was restricted to warmer northern regions (Layland, Upton, & Brown, 1994). However, *T. palmi* in temperate regions in Japan and China occurs in greenhouses, where it has become one of the most severe pests of vegetables (Kawai, 1990; Reitz et al., 2011). Spatial distribution of *T. palmi* expanded northward rapidly in recent years in China in greenhouses. With clear historical records and sensitivity to climatic variables, *T. palmi* is an ideal species to examine the effects of greenhouse environments on genetic structure.

In this study, we examined the population genetic structure of *T. palmi* across China and tested forces that shape this structure. Based on its tropical origin and stepping‐stone dispersal into temperate region, we hypothesized a structure formed by neutral processes such as genetic drift under geographic isolation. Based on sporadic colonization and the likelihood of strong selection by novel environmental conditions in temperate regions, we also hypothesized that greenhouse conditions should influence genetic structure of *T. palmi* as reflected by levels of diversity and genetic divergence. By understanding population genetic variation of *T. palmi* as well as evolutionary processes affecting it, factors that facilitate the spread of this invasive species and its potential to become a wider pest can be identified. This is one of the few studies that focus on population genetic structure of an agriculture insect pest in greenhouse conditions. The results shed light on understanding the impact of artificial environments on population genetic structure of pests more generally.

## 2 MATERIALS AND METHODS

### 2.1 Sample collection and DNA extraction

**Figure 1:**
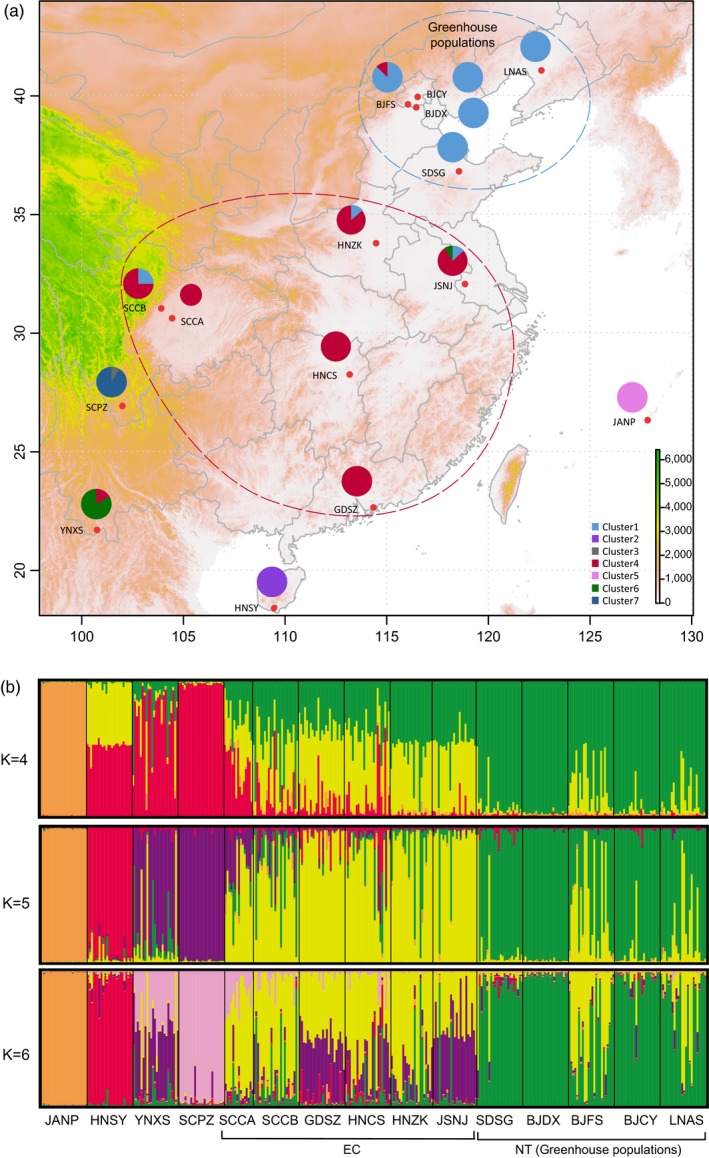
Sampling locations (red points in figure a) and population genetic structure of the 15 *Thrips palmi* populations inferred from BAPS analysis (pie charts in figure a) and STRUCTURE (b) based on 26 microsatellite markers. In the BAPS analysis, distributions of the seven identified clusters are shown by different colors. The color proportions in the pie charts represent the frequency of each cluster in a population. The red circle shows the central and eastern group (CE), and the blue circle shows the northern group (NT) of populations collected from greenhouses (a). In the STRUCTURE analysis, the optimal *K* determined based on delta *K* was 4. Clusters of individuals when *K* is 4, 5, and 6 are presented. Four outlier populations of JANP, HNSY, SCPZ, and YNXS, as well as two population groups of EC and NT, are presented. Codes for collection sites are shown in Table 1

We collected specimens of *T. palmi* from 14 geographic populations on eggplants and cucumbers across areas of its distribution in China and one population from Japan (Table 1, Figure 1a). Specimens were collected from 20 to 30 points in a field or greenhouse, separated by a distance of about 5–10 m. One individual was used from each collection point to reduce the likelihood of genotyping siblings. Populations from northern China were collected from greenhouses, where *T. palmi* is not known to overwinter in fields, while populations from southern China were collected from fields. In total, 348 female individuals from 15 populations were used for genotyping. We used female individuals for population genetic analysis due to the haplodiploid sex determination of *T. palmi*, in which haploid individuals develop to males while diploid individuals develop to females. Genomic DNA was extracted for each specimen with a DNeasy Blood & Tissue Kit (Qiagen).

**Table 1:** Collection information for specimens of *Thrips palmi* used in the study

Code	Collection location	Longitude (E)	Latitude (*N*)	Collection date	Host plant	Habit	No.
JANP	Japan, Okinawa	127°49'59.99"	26°19'59.99"	Jan/2016	Cucumber	Field	24
HNSY	Hainan Province, Sanya	109°27'32.70"	18°18'19.61"	Mar/2018	Eggplant	Field	24
YNXS	Yunnan Province, Xishuangbanna	100°45'4.34"	21°42'17.11"	Apr/2018	Eggplant	Field	24
SCPZ	Sichuan Province, Panzhihua	102°0'2.61"	26°55'57.89"	May/2016	Eggplant	Field	24
SCCA	Sichuan Province, Chengdu	104°26'35.55"	30°37'51.37"	Jul/2017	Eggplant	Field	15
SCCB	Sichuan Province, Chengdu	103°54'27.15"	31°2'42.66"	Aug/2018	Eggplant	Field	24
GDSZ	Guangdong Province, Shenzhen	114°20'51.70"	22°39'26.86"	Apr/2018	Eggplant	Field	24
HNCS	Hunan Province, Changsha	113°10'37.19"	28°15'31.19"	Jul/2017	Eggplant	Field	24
HNZK	Henan Province, Zhoukou	114°28'41.11"	33°47'37.07"	Sep/2017	Cucumber	Field	22
JSNJ	Jiangsu Province, Nanjing	118°50'56.17"	32°4'18.35"	Jul/2017	Eggplant	Field	23
SDSG	Shandong Province, Shouguang	118°33'14.19"	36°49'11.09"	May/2017	Cucumber	Greenhouse	24
BJDX	Beijing, Daxing district, Yufa town	116°20'9.79"	39°31'0.78"	Jul/2017	Eggplant	Greenhouse	24
BJFS	Beijing, Fangshan district	116°2'48.42"	39°38'42.82"	Jun/2016	Eggplant	Greenhouse	24
BJCY	Beijing, Chaoyang district	116°31'18.10"	39°57'32.23"	Oct/2017	Eggplant	Greenhouse	24
LNAS	Liaoning Province, Anshan	122°36'27.62"	41°4'31.80"	Sep/2018	Eggplant	Greenhouse	24

Note

Except for JANP, all other populations are from China. No., the number of individuals used for microsatellite genotyping and mitochondrial *cox1* sequencing.

### 2.2 Microsatellite genotyping and DNA sequencing

Twenty‐six genome‐wide microsatellite loci for *T. palmi* were used for genotyping (Table S1). PCR products were labeled by fluorescence for length determination with the method of Blacket, Robin, Good, Lee, and Miller (2012). Conditions for PCR amplification were described in previous publications (Cao, Li, et al., 2016; Song, Cao, Wang, Li, & Wei, 2017; Wang, Cao, Zhu, & Wei, 2016). PCR products were analyzed using ABI 3730xl DNA Analyzer (Applied Biosystems) with the GeneScan 500 LIZ size standard (Applied Biosystems) by Tsingke Biotechnology Co. Ltd. Microsatellite loci were genotyped using GENEMAPPER 4.0 (Applied Biosystems) and checked for stuttering and large allele dropout using MICRO‐CHECKER version 2.2.3 (van Oosterhout, Hutchinson, Wills, & Shipley, 2004).

To characterize mitochondrial variation and validate correct identification of the specimens, we sequenced a fragment of *cox1* gene on DNA barcoding region of animals by the primer pairs FO‐AF (5′ TTTCGTCTAACCATAAAGATATCGG 3′) and FO‐AR (5′ TAAACTTCTGGGTGCCCAAAAAATCA 3′) modified from previously published primers (Folmer, Black, Hoeh, Lutz, & Vrijenhoek, 1994) based on the mitochondrial genome sequence of *Franklienella occidentalis* (Yan et al., 2012). Polymerase chain reaction (PCR) was conducted using the Mastercycler pro system (Eppendorf) under standard conditions with an annealing temperature of 52°C. PCR components were added as recommended by the manufacturer of Takara LA Taq (Takara Biomedical). Amplified products were purified and sequenced on an ABI 3730xl DNA Analyzer by Tsingke Biotechnology Co. Ltd.

### 2.3 Genetic diversity analysis

For microsatellite loci, null allele frequency was estimated using FreeNA with 10,000 bootstraps (Chapuis & Estoup, 2007). Then, we examined Hardy–Weinberg equilibrium (HWE) for each locus at each population and linkage disequilibrium between each pair of loci at each population by GENEPOP version 4.2.1 (Rousset, 2008). Heterozygosity excess and deficit were tested by GENEPOP version 4.2.1. GENCLONE version 2.0 (Arnaud‐Haond & Belkhir, 2007) was used to estimate the total number of alleles (*A*_T_) and the unbiased expected heterozygosity (*H*_ET_) (Nei, 1978). Furthermore, we compared the number of alleles (*A*_S_) and standardized expected heterozygosity (*H*_ES_) among samples with different sample sizes using a rarefaction method in GENCLONE. Allelic richness (*A*_R_) and allelic richness of private alleles (*P*_AR_) were calculated with a rarefaction approach in HP‐RARE version 1.1 (Kalinowski, 2005) on a minimum sample size of 15 diploid individuals.

### 2.4 Population genetic structure analysis

For microsatellite loci, we used two Bayesian cluster methods and discriminant analysis of principal components (DAPC) to investigate genetic structure across the populations.

The first Bayesian method, Bayesian analysis of population genetic structure (BAPS), was conducted using BAPS version 6.0 (Cheng, Connor, Sirén, Aanensen, & Corander, 2013). This method can infer the clustering of individuals by incorporating spatial information. The maximum number of genetically diverged clusters (*K*) was set to 5, 8, 10 or 14, to ensure convergence and consistency of the results. For each *K*, 15 repeat runs were performed.

The second Bayesian model‐based clustering method was implemented in STRUCTURE version 2.3.4 (Pritchard, Stephens, & Donnelly, 2000). This method can estimate the ancestral gene frequencies and the admixture proportions for each individual (Lawson, van Dorp, & Falush, 2018). An admixture model with correlated allele frequencies was chosen. Thirty replicates for each *K* (from 1 to 10) were run with 200,000 Markov Chain Monte Carlo (MCMC) iterations after a burn‐in of 100,000 iterations. The optimal value of *K* was determined using the Delta (*K*) method (Evanno, Regnaut, & Goudet, 2005) by submitting the outputs of STRUCTURE to STRUCTURE HARVESTER WEB version 0.6.94 (Earl & Vonholdt, 2012). The membership coefficient matrices (Q‐matrices) of replicated runs for each *K* were combined using CLUMPP version 1.1.2 (Jakobsson & Rosenberg, 2007) with the Greedy algorithm and then visualized using DISTRUCT version 1.1 (Rosenberg, 2004).

Third, discriminant analysis of principal components (DAPC) was performed using adegenet version 2.0.1 (Jombart, 2008) in the R environment to identify the number of different genetic clusters. This method does not rely on biological models that can be a complementary analysis of model‐based methods such as BAPS and STRUCTURE.

For mitochondrial DNA, haplotypes and their distribution in each population were analyzed in DnaSP version 5.10 (Librado & Rozas, 2009). Phylogenetic relationships among mitochondrial *cox1* haplotypes were constructed using a split networking method implemented in SPLITSTREE version 4.13.1 (Huson & Bryant, 2006) with the neighbor‐net method under a distance model of K2P after 1,000 bootstraps.

### 2.5 Gene flow analysis

Recent and historical gene flow among populations was estimated using Bayesian methods implemented in BayesAss version 3.0.4 (Wilson & Rannala, 2003) and Migrate version 3.7.2 (Beerli & Felsenstein, 2001), respectively. BayesAss was modeled to estimate gene flow for the past 1–2 generations. We ran 100 million steps with different start seeds after preliminary runs for adjustment of mixing parameters for allele frequencies and inbreeding coefficients. The trace outputs of ten longer runs were combined using Tracer 1.6 (Rambaut, Drummond, Xie, Baele, & Suchard, 2018) to calculate mean migration with a burn‐in of 50 million. Migrate was modeled to estimate gene flow in all past times after the split of two populations. Mutation‐scale effective population size (*θ* = Neμ) for each population and mutation‐scale migration rate (*M* = m/μ) among all populations, where μ is the mutation rate of genetic markers per generation, were simultaneously estimated with the Bayesian search strategy in Migrate version 3.7.2. Parameter values were as follows: long‐chains = 1, long‐inc = 20, long‐sample = 100,000, burn‐in = 100,000, heating = YES:1:(1.0:1.5:3.0:10,000.0), heated‐swap = YES, and replicate = YES:4. In the first run, θ and *M* were estimated from *F*_ST_ values, while in subsequent runs, Bayesian estimates of θ and *M* from the previous run were used.

### 2.6 Demographic history analysis

We estimated the variation of effective population size for each population based on microsatellite loci using coalescent algorithms implemented in Migraine version 0.5.4 (Rousset, Beeravolu, & Leblois, 2017). This software uses PAC‐likelihood (product of approximate conditional likelihoods) based on quantities inherent to the importance of sampling algorithms (Cornuet & Beaumont, 2007). Since inferences under maximum‐likelihood models are very sensitive to mutational processes (Leblois et al., 2014), we chose 22 perfect loci with identical repeat motif for analysis. A generalized stepwise model (GSM) was used for microsatellite mutation. First, we estimated the pGSM parameter for the GSM model and mutation‐scaled effective population sizes (*θ*) for current population (2Nμ) in a single population model (OnePop). Then, we used a single population model with a single continuous past variation in population size (OnePopVarSize) to estimate mutation‐scaled effective population sizes (*θ*) for the current population (2Nμ) and ancestral population (2Nancμ) with fixed pGSM as estimated in previous steps. For both demographic models, we first conducted two short iterations with 800 points and 200 runs per point to generate preliminary points. Then, 15 iterations with 800 points and 1,000 runs per point were conducted starting from points of last iteration. In the third round, 3 iterations with 800 points and 20,000 runs per point were conducted starting from points of the last iteration. Estimation of past demographic changes is usually challenging (Beaumont, 2010; Hey, 2010; Rousset et al., 2017). When the OnePopVarSize model was used, estimation became instable. We evaluated the confidence of OnePopVarSize‐based estimation by comparing the similarity of 2Nμ values estimated by OnePop and OnePopVarSize as well as the confidence intervals of the estimates.

### 2.7 Partitioning geographic and climate effects on genetic variation

We analyzed geographic/climatic effects on population genetic variation using two methods. First, isolation by distance (IBD) was tested in samples from China by correlating pairwise genetic differentiation (estimated as *F*_ST_/(1−*F*_ST_)) with geographic distance using the package ade4 in R, with 10,000 permutations. Pairwise *F*_ST_ values between populations based on microsatellites were calculated with GENEPOP version 4.2.1 (Rousset, 2008).

Second, we used a multivariate approach of redundancy analysis (RDA) to estimate the extent to which the variance in microsatellite genotypes was explained by climate and geography and by their collinear portion (spatially autocorrelated climatic variation). RDA is a constrained linear ordination method that combines multiple linear regression and PCA (principle component analysis). For climate data, we obtained 19 climate variables for each collection site from worldclim.org. To account for the influence of greenhouse conditions, we added variable of habitat with two values of greenhouse and field. Variables of climate and habitat were classified as environmental conditions. Geographic distances among 15 populations reflected isolation‐by‐distance effects on population structure. The matrices of pairwise geographic distances were transformed into principal components of neighborhood matrices (PCNM) using the function *pcnm* in *vegan* R package (https://github.com/vegandevs/vegan). Only the first half of positive eigenvectors was retained as explanatory variables of population structure. To avoid high collinearity, we excluded variables with a variance inflation factor (VIF) over 10. We conducted a series of RDAs initially with all variables in the first model, removing a variable with the highest VIF each round, and stopping when all VIF values were below 10. Four PCNM vectors, six environmental variables of habitat and five climate variables remained including three temperature‐related (bio3, isothermality; bio5, max temperature of warmest month; bio8, mean temperature of wettest quarter) and two precipitation‐related (bio15, precipitation seasonality; bio18, precipitation of warmest quarter) variables. Both full (environmental and geography) and partial (environmental or geography) models of RDA were analyzed in the R package *vegan*. The independent effect of environment was the variance values for the constrained matrix of geography in the appropriate partial model, while the independent effect of geography was the equivalent for the constrained matrix of environment. The collinear proportion was calculated by subtracting the independent effects of environment and geography from the total amount of variance explained in the full RDA model.

## 3 RESULTS

### 3.1 Population genetic diversity

Among 26 microsatellite loci, 305 of the 4,875 locus–locus pairs in a population (of which 159 of 325 came from the Japanese population) showed linkage disequilibrium (*p* < .01), while three of 325 locus pairs across all populations showed linkage disequilibrium. Thirty‐seven of 390 loci‐population pairs deviated from HWE (*p* < .01). However, no locus pair was linked and no locus deviated from HWE in all populations. The Japanese population (JANP) showed the lowest level of genetic diversity based on all parameters. For Chinese populations, two southwestern populations showed the highest level of genetic diversity, five population from central areas showed medium levels of diversity, whereas five northern populations collected from greenhouses and one island population from southern China showed the lowest levels. Similar results were found in estimations based on mitochondrial gene sequence (Table 2).

**Table 2:** Genetic diversity of the 15 populations of *Thrips palmi* based on 26 microsatellite loci and mtDNA

Population	Diversity level	Microsatellites								Mitochondrial DNA		
		*H*_O_	*H*_ET_	*H*_ES_	*F*_IS_	*A*_R_	*A*_T_	*A*_S_	*P*_AR_	*H*	*h*	*π*
JANP	Very low	0.2803	0.3208	0.3213	−0.0769	1.91	67	62.62	0.09	1	0.00	0.0000
HNSY	Low	0.5050	0.6362	0.6360	0.0195	4.20	192	158.97	0.32	5	0.32	0.0011
YNXS	High	0.6174	0.7211	0.7209	0.0844	6.31	240	201.66	0.63	5	0.44	0.0044
SCPZ	High	0.5545	0.7829	0.7817	0.1840	6.48	297	241.68	0.97	3	0.30	0.0030
SCCA	Medium	0.6491	0.7423	0.7423	0.0408	5.87	209	209.00	0.26	4	0.61	0.0030
SCCB	Medium	0.5848	0.7089	0.7076	0.1282	5.21	210	176.91	0.08	3	0.24	0.0006
GDSZ	Medium	0.5719	0.6439	0.6432	0.0819	5.16	185	156.83	0.24	3	0.22	0.0006
HNCS	Medium	0.5901	0.6771	0.6762	0.0479	5.10	209	177.32	0.13	5	0.64	0.0020
HNZK	Medium	0.5774	0.6630	0.6629	0.0649	4.84	196	168.90	0.17	1	0.00	0.0000
JSNJ	Low	0.5437	0.6459	0.6452	0.0908	4.31	170	148.19	0.19	2	0.29	0.0007
SDSG	Low	0.5773	0.6223	0.6214	0.0471	4.64	170	144.82	0.05	2	0.29	0.0007
BJDX	Low	0.5737	0.5956	0.5953	0.0375	4.12	122	114.16	0.04	1	0.00	0.0000
BJFS	Low	0.5776	0.6340	0.6330	0.0587	4.56	157	138.01	0.02	1	0.00	0.0000
BJCY	Low	0.5657	0.6574	0.6566	0.0447	4.11	157	138.42	0.05	2	0.23	0.0006
LNAS	Low	0.5952	0.6219	0.6215	0.0366	4.57	149	131.63	0.04	1	0.00	0.0000

Note

The diversity level was classified based on the overall genetic diversity of all estimated parameters. The last five populations were collected from greenhouses.

Abbreviations: *π*, nucleotide diversity; *A*_R_, average allelic richness; *A*_S_, standardized total number of alleles for 15 specimens per samples; *A*_T_, total number of alleles; *F*_IS_, inbreeding coefficient; *h*, haplotype diversity; *H*, number of haplotype; *H*_ES_, standardized expected heterozygosity (for 15 specimens); *H*_ET_, expected heterozygosity; *H*_O_, observed heterozygosity; *P*_AR_, private allelic richness.

### 3.2 Population genetic structure

*F*_ST_ values indicated that three populations (JANP, HNSY, SCPZ) showed relatively high genetic differentiation from the other populations (Table 3). BAPS analysis divided all individuals into seven clusters. Two clusters were mainly distributed in two island populations (JANP and HNSY), and four were in southwestern populations from China (YNXS and SCPZ). The northern populations were mainly composed of one cluster (Figure 1a, cluster 1 in light blue), whereas central and eastern populations were mainly composed of another cluster (Figure 1a, cluster 4 in red). STRUCTURE analysis indicated that the optimal cluster with all individuals included was four (Figure 1b). Except for the JANP population, three other population groups were identified in the STRUCTURE analysis, corresponding to results from the BAPS analysis. Two populations (YNXS and SCPZ) were classified into a southwestern group, six populations were placed into an eastern and central group, and five populations were placed into a northern group. Apparent admixture was found in populations from the eastern and central groups and two populations from the northern group. DAPC analysis showed two islands (JANP and HNSY) and two southwestern (YNXS and SCPZ) populations as outliers; most populations were clustered according to their geographic distributions (Figure S1). Overall, population genetic structure identified four differentiated populations and two population groups corresponding to their geographic distribution.

**Table 3:**
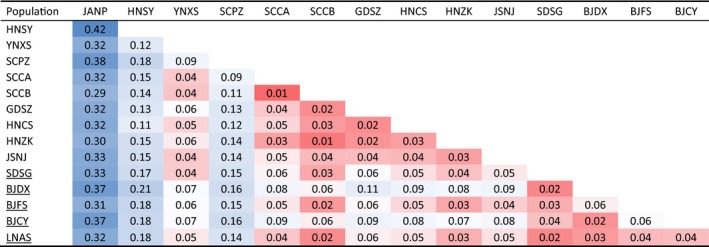
Pairwise *F*_ST_ among 15 populations of *Thrips palmi*

Note

All values are statistically significant under *p* = .01. Values were shaded from high to low by deep blue to deep red. Five populations collected from greenhouses are underlined.

### 3.3 Mitochondrial haplotype distribution and phylogeny

**Figure 2:**
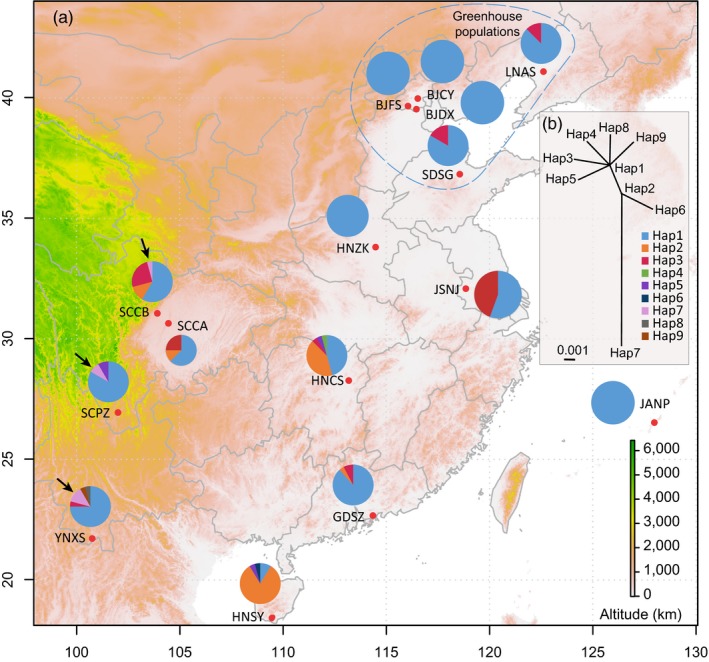
Distribution of the mitochondrial *cox1* haplotypes in each population (a) and split networks of the haplotypes inferred from SPLITSTREE (b). Different colors in the pie charts indicate the proportion of haplotypes in each population. The populations in the blue circle were collected from greenhouses. The three black arrows point to six individuals with the genetically distant haplotype of Hap7 in three southwestern populations of YNXS, SCPZ, and SCCB

Seven mitochondrial *cox1* haplotypes were identified among all individuals (Figure 2a). Distributions of the haplotypes showed a clear geographic pattern. The most diverged Hap7 (Figure 2b) was exclusively found in three southwestern populations (arrows in Figure 2a); Hap1 was mostly found in northern populations, while Hap3 was mostly found in populations collected from eastern and central areas of China (Figure 2a).

### 3.4 Gene flow and effective population size

BayesAss did not detect gene flow among populations in recent generations. Migrate analysis identified migration among populations historically. When we calculated the scaled gene flow per generation, there was no clear pattern of gene flow, except for low values from and to the two island populations of JANP and HNSY (Table 4).

**Table 4:**
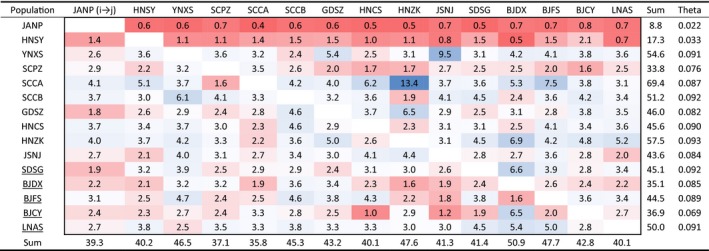
Historical effective number of migrants per generation among populations and effective population size (Theta) of *Thrips palmi*

Note

The effective number of migrants per generation is shaded from high to low by deep blue to deep red. Five populations collected from greenhouses are underlined.

Among the 15 populations, seven showed an expansion of effective population size, two showed a reduction (JANP and GDZD), and six showed no significant difference between current and ancestral effective population sizes (Table 5). Based on our criteria, estimates using OnePopVarSize model fitted well in at least five populations, three of which showed population expansion. Three of the five greenhouse populations showed population expansion (Table 5).

**Table 5:** Estimated population size of *Thrips palmi* populations estimated by the maximum‐likelihood method implemented in Migraine

Population	OnePop model		OnePopVarSize model		Population size variation
	pGSM	2Nμ	2Nμ	2N_anc_μ	
JANP	0.59 [0.440–0.719]	0.24 [0.146–0.369]	0.09 [0.0001–0.26]	**0.76 [0.212–27.53]**	Reduction
HNSY	0.59 [0.496–0.671]	1.37 [1.051–1.748]	6.06 [1.896–108.6]	**0.92 [0.559–1.359]**	Expansion
YNXS	0.52 [0.428–0.592]	3.59 [2.844–4.509]	6.00 [3.660–1484]	**1.88 [0.068–3.237]**	Expansion
SCPZ	0.54 [0.446–0.616]	2.65 [2.028–3.441]	4.04 [2.813–14.21]	0.00 [0.0004–2.72]	Expansion
SCCA	0.52 [0.440–0.598]	3.16 [2.879–4.532]	5.86 [0.00001‐NA]	3.07 [NA−848.600]	Expansion
SCCB	0.44 [0.348–0.532]	2.46 [1.913–3.176]	5.78 [0.187–18.19]	2.25 [0.0008–202.1]	No. sign.
GDSZ	0.50 [0.409–0.587]	2.17 [1.695–2.757]	1.33 [0.907–1.356]	20.61 [18.180–21.180]	Reduction
HNCS	0.48 [0.383–0.562]	2.10 [1.631–2.682]	2.57 [1.682–3.163]	0.70 [NA‐NA]	No. sign.
HNZK	0.52 [0.434–0.606]	1.86 [1.430–2.399]	3.53 [0.0248 ‐ NA]	**1.39 [0.0499–18.730]**	No. sign.
JSNJ	0.58 [0.489–0.659]	1.34 [1.012–1.744]	5.38 [2.883–10.71]	7.59 [5.791–19.430]	No. sign.
SDSG	0.53 [0.431–0.617]	1.64 [1.258–2.110]	1.08 [0.063–8.706]	1.96 [0.0003‐NA]	No. sign.
BJDX	0.56 [0.470–0.650]	1.22 [0.916–1.605]	2.94 [2.380–3.639]	2.97 [2.673–4.127]	No. sign.
BJFS	0.49 [0.389–0.575]	1.73 [1.331–2.239]	2.19 [2.118–4.743]	0.06 [0.054–0.063]	Expansion
BJCY	0.54 [0.447–0.631]	1.30 [0.980–1.701]	0.60 [0.471–0.671]	0.23 [0.210–0.302]	Expansion
LNAS	0.51 [0.410–0.598]	1.74 [1.331–2.249]	6.37 [5.567–7.163]	**1.65 [1.630–2.247]**	Expansion

Note

95% of coverage confidence interval are provided in bracket; pGSM, parameter for GSM model of microsatellite mutation; 2Nμ, the current effective population size scaled by mutation rate; 2N_anc_μ, ancestral effective population size scaled by mutation rate; type of population size variation was determined by overlap of 95% of coverage confidence interval between 2Nμ and 2N_anc_μ estimated by OnePopVarSize model. The bolded and underlined numbers show confidence estimation from OnePopVarSize model determined based on differences between estimation of 2Nμ by two models and confidence intervals of estimated 2N_anc_μ. Five populations collected from greenhouses are underlined. NA, indicates the value is either extremely low or high.

### 3.5 Geographic and climate effects on genetic variance

**Figure 3:**
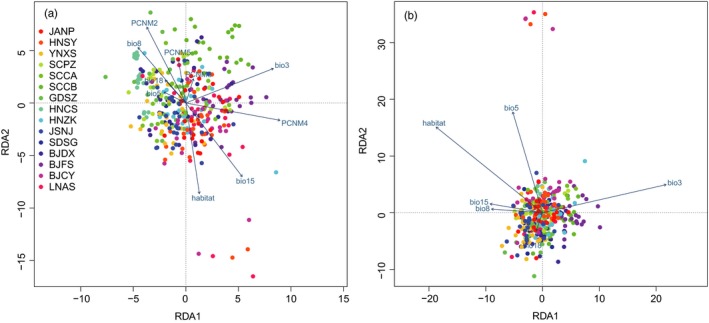
RDA analysis on genetic variance explained by the environmental effects of climate and habitat. A full RDA model was run by considering environmental and geographic effects simultaneously (a), and a partial RDA model was run by constraining geographic effects to analyze the correlation of environmental variables (b). Individuals from the same population are indicated by circles with the same color. PCNM2‐5, geographic variables; bio3, isothermality; bio5, maximum temperature of warmest month; bio8, mean temperature of wettest quarter; bio15, precipitation seasonality; bio18, precipitation of warmest quarter; habitat, samples collected from greenhouse or field. Correlations of each variable are indicated by an arrow. Long arrows indicate a high correlation between the variable and genetic distance

A Mantel test indicated a significant correlation between genetic distance and geographic distance (*r* = .6305, *p* = .001). The effects of geography, the environment, and their interaction explained 17.9% of the total genetic variance. For the explained genetic variance, independent effects of environmental conditions (including climatic variables and habitat, i.e., greenhouse or field) and geography accounted for 51.68% and 32.06%, respectively, while their collinear component accounted for 16.26%. When environmental and geographic effects were considered simultaneously in the RDA analysis, habitat, three climatic variables (isothermality [bio3], precipitation seasonality [bio15], and mean temperature of wettest quarter [bio8]), and two geographic variables (PCNM2 and PCNM4) were highly correlated with genetic distance (Figure 3a). When geographic variables were constrained in the RDA analysis, habitat, isothermality of temperature (bio3), and maximum temperature of warmest month (bio5) were highly correlated with genetic distance (Figure 3b).

## 4 DISCUSSION

### 4.1 Origin of Thrips palmi

Population genetic analyses provide novel approaches for investigating dispersal and invasion routes of species compared to historical records (Boissin et al., 2012; Estoup & Guillemaud, 2010; Lombaert et al., 2014; Rollins, Woolnough, Wilton, Sinclair, & Sherwin, 2009). In *T. palmi*, we showed that populations from southern China exhibited high levels of genetic differentiation from other populations as well as high genetic diversity, while the northern populations showed low genetic differentiation and diversity, suggesting that *T. palmi* has recently dispersed to northern China. This is congruent with worldwide historical records showing that this species originated from tropical counties of Asia and spread into pantropical regions in its early stages of range expansion (Cannon et al., 2007).

### 4.2 Population processes shaping the genetic structure of Thrips palmi

We tested possible population processes that may shape the genetic structure of *T. palmi* in China. The presence of isolation by distance and absence of recent gene flow suggests that genetic drift plays an important role in genetic differentiation. While the introduction sources of *T. palmi* in southern China are unclear in the absence of reference populations from its native range, it appears that at least some of the northern populations in China have come from a single source.

Size of founder populations may influence the future response of invasive species in newly introduced areas (Signorile et al., 2014). We estimated the effective size of current (2Nμ) and ancestral (2N_anc_μ) populations for each population. There was no significant difference in 2N_anc_μ among the five well‐estimated populations, ranging on average from 0.76 to 1.88, indicating that demographic history may not have had much impact on population differentiation.

### 4.3 Greenhouse‐related population structure and climatic adaptation in Thrips palmi

The genetic structure of *T. palmi* populations in China follows geography, whereas five populations collected from greenhouses in northern China form a genetic group as inferred from the microsatellites. This pattern suggests little impact of geography on genetic structure across the greenhouses. Genomic SNPs are really needed to provide information on genes that may be related to climatic adaptation (Janes et al., 2014) as well as adaptation to the greenhouse environment. Stable microsatellite loci such as developed here at a genome‐wide scale can provide information of adaptation based on linkage to selected loci; however with a limited number of microsatellites, our interpretations of climate‐related patterns should be viewed as indicators of how climate factors might influence patterns of overall population genetic structure through factors such as generation time, drift, and movement pathways.

Our RDA analysis showed that environmental conditions could explain more than half the explained variation in genetic distances among the populations, or 9.25% of total variation in *T. palmi*, suggesting environmental impacts on geographic structure. Among the variables used, habitat as well as isothermality of temperature (bio3) and maximum temperature of warmest month (bio5) are top variables that contributed to the genetic variation across sites. These results suggest that population genetic structure of *T. palmi* is related to greenhouse conditions and climate. Environmental conditions may impose direct strong selection pressures on *T. palmi* as evident from biological studies across the natural distribution range of this species (McDosald et al., 1999). As a tropical species, *T. palmi* cannot easily deal with low temperatures. The threshold for the development of *T. palmi* from egg to adult is about 10–11°C, and a sum of effective temperatures is 189–194 degree‐days (Kawai, 1985; McDosald et al., 1999). Based on limited temperature adaptation, introduced *T. palmi* were successfully eradicated in England after they were introduced (MacLeod, Head, & Gaunt, 2004). In northern Japan, the species could not overwinter outdoors and survived cold winters in greenhouses. The population dynamics of *T. palmi* can also be influenced by humidity (Su, Chiu, & Lin, 1985). In Australia, the distribution of *T. palmi* is restricted to northern regions with warm temperatures in winter, and its southern distribution may be limited by prevailing aridity (Layland et al., 1994).

These limits point to the likelihood of migration and population size of the thrips being limited by environmental conditions in China. Greenhouse conditions in northern China can help *T. palmi* to persist despite low temperatures outside, and they also provide humid conditions. The ecological opportunity provided by greenhouses may explain why there was a signature of populations expanding in three of five greenhouse populations. Such ecological opportunities could promote adaptive radiation by generating genetic changes in organisms (Stroud & Losos, 2016; Yoder et al., 2010). Further studies involving well‐designed experiments are needed to explore this from a biological and genomic perspective.

### 4.4 Distinct genetic structure between two thrips species

Thrips are tiny insects that are difficult to detect in quarantine. Invasion and dispersal of such small insects are usually mediated by human activities such as plant transport. Both *F. occidentalis* and *T. palmi* are serious invasive pest of agriculture. Nevertheless, our studies showed distinct genetic structure in these two thrips across their distribution range of China. The *F. occidentalis* thrips showed population genetic differentiation unrelated to its geographic distribution in China, pointing to multiple introductions and human‐mediated dispersal in sporadic directions (Cao et al., 2017). In contrast, in *T. palmi* there was a high level of genetic differentiation across its spatial distribution in China‐related partly to geographic distance. This pattern of population genetic structure is similar to that found in species with stepping‐stone dispersal (Cao, Wei, Wei, Hoffmann, Wen, & Chen, 2016; Kimura & Weiss, 1964; Wei et al., 2015) rather than sporadic long‐distance dispersal (Cao et al., 2017). The pattern is also congruent with historical records in China; *T. palmi* was first reported in southern China and occurred in northern areas much later (Yi & Liang, 2001; Zhang, Han, & Fu, 1985). *Thrips palmi* is particularly abundant on the foliage of cucumbers and eggplants, and dispersal of this thrips may have been slow because these vegetables are transported as fruit rather than foliage (Huang, 1989).

Distinct genetic structures and different invasion patterns likely reflect differences in the biology of the two thrips species. A temperate origin may allow *F. occidentalis* to invade a wider range of environmental conditions than *T. palmi* which originated from the tropics. It is likely that *F. occidentalis* is pre‐adapted to newly introduced areas and a limited number of individuals may then be sufficient to establish a population. On the other hand, more individuals may be needed for adaptation in *T. palmi* to provide abundant genetic variation on which natural selection can act. This is consistent with the similar levels of genetic diversity among population of *T. palmi* when compared to the different levels of genetic diversity among populations of *F. occidentalis* (Table 2 in this study and Cao et al., 2017).

### 4.5 Implications for pest management

Greenhouses extend the ability to produce crops in different seasons and regions. The protected conditions in greenhouses lead to serious pest problems that are different from outdoor fields, such as outbreaks of whiteflies, mites, and thrips (Gerson & Weintraub, 2012). Our findings illustrate that they can also influence the genetic structure and variation of the pest organism through affecting population processes. These, in turn, could influence processes like the evolution of pesticide resistance that are influenced by climatic conditions (Maino, Kong, Hoffmann, Barton, & Kearney, 2016; Wimmer, Hoffmann, & Schausberger, 2008). Compared to the temperate species *F. occidentalis*, which can be readily dispersed across a wide range through human activities, dispersal of the tropical species into temperate areas will be restricted by environmental conditions. However, environmental restrictions can be overcome by using artificial environments and there may also be evolutionary adaptation to the relatively constant conditions present in glasshouses. Thus, evolutionary adaptation by *T. palmi* should be further considered both to cold conditions and to the relatively constant conditions of greenhouses given that adaptive shifts can influence predictions around pest distributions and pest abundance.

## 5 CONCLUSIONS

In this study, we revealed population genetic structure and potential evolutionary forces affecting genetic differentiation of an invasive agricultural pest, *T. palmi*. We found that environmental factors and geographic isolation correlated with genetic differentiation. The ecological opportunity provided by greenhouses may contribute to recently expanded populations of *T. palmi*.

## CONFLICT OF INTEREST

None declared.

## Data Availability

All data were achieved in Dryad under DOI: https://doi.org/10.5061/dryad.bp27sd7 (Cao et al., 2019).
