# Supplementary material for: Population analysis reveals genetic structure of an invasive agricultural thrips pest related to invasion of greenhouses and suitable climatic space
Source: Evol Appl. 2019 Aug 8;12(10):1868–80. doi: 10.1111/eva.12847 (PMC6824073; doi:10.1111/eva.12847)
Supplement: Supplementary file 1 [file EVA-12-1868-s001.docx]

| Table S1 Twenty-six microsatellite markers developed for *Thrips palmi* | | | | | | | |
| --- | --- | --- | --- | --- | --- | --- | --- |
| **Locus** | **Dye** | **Repeat Motif** | **Allele Number** | **Size range (bp)** | **PCR product size** | **Forward primer (5'-3')** | **Reverse primer (5'-3')** |
| TP4-S02 | HEX | (ACAG)_7_ | 5 | 111-127 | 112 | CCGTCCGTGTCGTATCTGAC | CCTGTCCGTCTGTCAGTGTC |
| TP5-S03 | HEX | (ACGGC)_8_ | 8 | 115-167 | 113 | CGCTCCTCATTCGCTCGTAT | TCGACTCGCCATTCATAGGC |
| TP4-S04 | HEX | (ACAG)_8_ | 5 | 136-154 | 126 | CAAGACCACGTTTGCCACTG | CCAATTGCCTCGCTGTTACG |
| TP4-S06 | HEX | (ACAG)_7_ | 12 | 154-212 | 139 | AAGGAGGCGAATCAAGCGAT | CTCGTCTCGACAAACGGTGA |
| TP4-S09 | HEX | (ACAG)_7_ | 5 | 153-173 | 155 | GCGATTTGCGTCCGATTTCA | AGCCAATACCTCTCGTGCTG |
| TP4-S11 | HEX | (AGCC)_12_ | 12 | 160-256 | 175 | TGTGGGTCTCAGGTAGCAGA | TACGCAGAGTGGAACGGAAC |
| TP4-S12 | HEX | (ACAG)_15_ | 5 | 157-198 | 177 | GGTTCACTAATCCTCCGCGT | CCGCCATTAGTGACTCTCCC |
| TP3-S17 | HEX | (ACC)_7_ | 8 | 207-271 | 201 | GTCACGTCCATCTACCACCG | CCTGCTGGAGACAACCACTT |
| TP3-S19 | HEX | (AGG)_8_ | 6 | 221-237 | 203 | TCGGGATTCGGACTGGTTTG | GACCTTGGTTGGCCTTGACT |
| TP3-S22 | FAM | (AAC)_8_ | 6 | 239-258 | 225 | GTAGAGCAGTGCATCGAGCT | ATCTGGGCACTCATTGGACG |
| TP3-S23 | FAM | (AAC)_12_ | 11 | 224-259 | 226 | GAAAGCCACGAGGAGATCCC | TTGACAGCGAAGTTTCCCGA |
| TP4-S24 | FAM | (ACAG)_19_ | 18 | 192-278 | 227 | CGCGTGCCAGATCAATGAAG | ATCGTCTCTGGACCGATTGC |
| TP4-S25 | FAM | (ACAG)_17_ | 7 | 197-245 | 228 | TGCATAACTCTGGGAGGCAC | TGATCCCTTGTCTCGGCCTA |
| TP4-S27 | FAM | (AGAT)_22_ | 11 | 218-266 | 236 | TATCGCGCTCTATGCTTCCC | CAGGGAGCTTGATGCAAGGA |
| TP4-S28 | FAM | (ACGG)_8_ | 5 | 259-276 | 241 | AAAGCCGAAGCCTCCATACC | CCGACCAACACAGCAACAAG |
| TP4-S29 | FAM | (AGGC)_11_ | 7 | 231-259 | 242 | GAGGCTCATTGCACGCAAAT | CCGGAGCGTGAGGAATTGTA |
| TP4-S30 | FAM | (AGGC)_10_ | 13 | 250-347 | 245 | CGGACGGACTCGCAATATGA | TTGTGGTCTTCTCCTTGGCC |
| TP3-S31 | FAM | (AGC)_9_ | 11 | 254-324 | 247 | GGCTCGTACTGTCTCACCAC | TCGCCGCTTCTTAGAACTCC |
| TP4-S32 | ROX | (ACAG)_12_ | 9 | 233-274 | 249 | TGGTCAAGCCAAGCGAATCT | GGCAATCACCAGGGAGGATC |
| TP3-S36 | ROX | (ACC)_7_ | 2 | 278-281 | 260 | GCCCTCTTCACTTCAGCACT | CCACCACACCGGAATATCCC |
| TP4-S40 | ROX | (ACAG)_11_ | 9 | 257-305 | 269 | CAAGGTGTGAAGGAAGCCGA | CGGGCTTCGATGAGATCCTC |
| TP4-S42 | ROX | (ACTC)_7_ | 2 | 279-291 | 271 | GCGATTGCCTCCGAGTTAGT | CCATGAACGTGTTGGCAGTG |
| TP3-S51 | ROX | (ACG)_7_ | 3 | 315-327 | 305 | GGAGCCCTTGGAGCGAATTA | GGAGCTATCTCGACGTTCCG |
| TP3-S56 | ROX | (ATC)_7_ | 3 | 348-354 | 332 | GGGATTTGACGGCAGAGCTA | GGTGGAGCTCGTTCAGAACA |
| TP4-S57 | ROX | (ATCC)_9_ | 14 | 320-409 | 335 | AGTGTAGGCGCTCTTTGCTT | GTGACTCTGTGACACGGCTT |
| TP4-S58 | ROX | (AAAG)_23_ | 16 | 320-457 | 341 | ACCGCCGTCTCTTGATAAGC | TCACCAGACGGATGCTTACG |


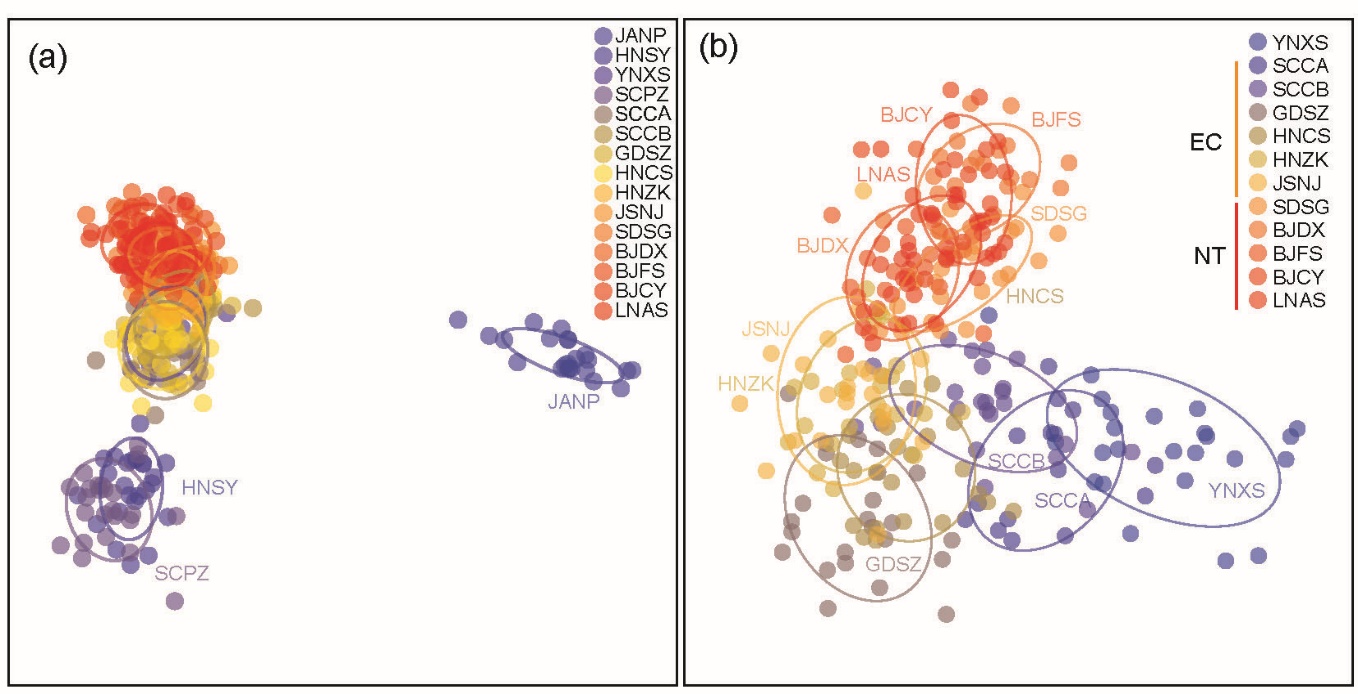


**Figure S1** Population structure of *Thrips palmi* inferred from DAPC analysis based on 26 microsatellite markers. Three outlier populations (JANP, HNSY and SCPZ) were identified when all populations were analyzed (a); when those outliers were excluded, the remaining populations were clustered corresponding to their geographical distributions forming eastern and central group (EC) and northern group (NT) (b). Codes for collection sites are shown in Table 1.
